# The French version of the HSCL-25 has now been validated for use in primary care

**DOI:** 10.1371/journal.pone.0214804

**Published:** 2019-04-04

**Authors:** Patrice Nabbe, Jean Yves Le Reste, Morgane Guillou-Landreat, Florence Gatineau, Bernard Le Floch, Tristan Montier, Harm Van Marwijk, Paul Van Royen

**Affiliations:** 1 EA 7479 SPURBO, Department of general practice, Université de Bretagne Occidentale, Brest, France; 2 EA 7479 SPURBO, Department of addictology, Université de Bretagne Occidentale, Brest, France; 3 Unité INSERM 1078, SFR 148 ScInBioS, Université de Bretagne Occidentale, Brest, France; 4 Division of Population Health, Health Services Research and Primary Care, School of Health Sciences, Faculty of Biology, Medicine and Health, The University of Manchester I Williamson Building, Oxford Road, Manchester, United Kingdom; 5 Department of Primary and Interdisciplinary Care, Faculty of Medicine and Health Sciences, Universiteit Antwerpen, Antwerpen, Belgium; Department of Psychiatry and Neuropsychology, Maastricht University Medical Center, NETHERLANDS

## Abstract

**Background:**

The Hopkins Symptom Checklist in 25 items (HSCL-25) helps to assess anxiety and depression in Primary Care. Anxiety and depression show considerable overlap in primary care. This self-administrated questionnaire is valid, reliable and ergonomic in the original US version. We have translated it into French. The aim of this study was to estimate the test characteristics of the HSCL-25, in its French version (F-HSCL-25), by comparing it to the Present State Examination-9 French version (F-PSE-9) and by determining its internal validity and dimensions.

**Method:**

Outpatients from three French General Practice settings (rural, semi-rural and urban) were recruited: approximately 20,000 outpatients among 17 GPs. Two groups were formed: F-HSCL-25 ≥1.75 and F-HSCL-25 <1.75. A validated cut-off score of > 1.75 was considered to indicate a clinically relevant level of symptoms of depression and anxiety. In order to obtain two balanced groups, a different method of randomization was chosen for each group. The F-PSE-9 was randomly administered to 1 in 2 patients in the F-HSCL-25 ≥1.75 group, and to 1 in 16 in the (much larger) F-HSCL-25 <1.75 group. The diagnostic performance was assessed and the test results obtained from both groups were compared with their F-PSE-9 results.

**Results:**

Of the 1126 patients who completed the F-HCL-25, 886 joined the F-HSCL-25 <1.75 group and 240 the F-HSCL-25 ≥1.75 group. The overall prevalence of depression, using the F-HSCL-25, was 21% in these medical practices. The diagnostic performance of the F-HSCL-25 versus the F-PSE-9, the external criteria were as follows: Positive Predictive Value (PPV) 69.8%, Negative Predictive Value (NPV) 87%; Sensitivity 59.1%, and Specificity 91.4%. The Principal Component Analysis showed that F-HSCL-25 is a one-dimensional tool (anxiety and depression dimensions combined) with a Cronbach Alpha of 0.93.

**Conclusion:**

The F-HSCL-25 is an appropriate diagnostic tool for anxiety and depression in primary care in France due to its high specificity and high NPV. The HSCL-25 scale has a high eigenvalue. This pilot study will be extended throughout Europe; however, preliminary evidence suggests that the HSCL-25 is a reliable and suitable diagnostic tool for primary care.

## Introduction

Major depression affects 4.4% of the world population [1]. Estimates of the prevalence in the general population vary in Europe but are currently around 6% to 18% [2]. Furthermore, the prevalence is twice as high for women [3]. A prevalence increase of more than 18% was observed between 2005 and 2015 [4]. Within the French population as a whole, the prevalence is estimated to be between 5% and 12% [5]. Currently, nearly 8 million French people have experienced, or will experience, depression during their lifetime [6]. Depression has a significant impact on emotional, social and occupational life and is a major risk factor for suicide [7].

The general practitioner (GP) diagnosis for major depression has a high specificity but a low sensitivity in routine care but, as GPs can also offer efficient follow-up, primary care is a good place to organize treatment [8]. This syndromic disorder is not easy to diagnose due to the wide variety of ways in which it may be presented [9]. In most European countries, GPs are the first, and often the only, physicians to take care of depressed patients but they generally have little time [10]. A fast, efficient and sensitive tool with a reasonable specificity and negative predictive value, would add value and save time, thereby improving performance management in primary care.

From the many diagnostic tools available for combined European research studies, the HSCL-25 has been selected, using a European consensus procedure, based on a systematic review of the literature. It combines high quality reliability, effectiveness and ergonomics with a conceptual connection to the formal diagnostic criteria of the DSM [11][12]. The HSCL-25 is a short-form diagnostic tool derived from the HSCL-90 [13][14][15][16]. This is a comprehensive, systematized, semi-directed, clinically self-administered questionnaire [15][16].

The specificity compared with clinical interview is robust: between 0.78 to 0.88, the reliability (Alpha de Cronbach) is between 0.87 to 0.97 [14]. The HSCL-25 short length self-administered format is perfectly suited for use in busy primary care settings with many competing demands. It may represent a practical instrument to alert French GPs to potentially depressive or anxious symptomatology.

The score is based on 25 questions divided into two sub-sections related to the presence and intensity of symptoms of depression and anxiety experienced during the previous week. Patients select one of the four responses for each item on a 4-point Likert scale, ranging from 1 (strongly disagree) to 4 (completely agree). Completing the questionnaire takes between 5 and 10 minutes. The final score is calculated by dividing the sum of the scores of all the items by 25 (the final score ranges from 1.00 to 4.00). A diagnosis of Major Depression, defined as "a case requiring treatment," is generally above a threshold of 1.75 [17].

The HSCL-25 was translated into French using a well-established procedure in primary care, involving a forward/backward translation based on a Delphi procedure, combined with a cultural check to maintain linguistic and semantic reliability (S1 Appendix) [18].

In 1993, Nettlebladt & al. evaluated the accuracy of the HSCL-25 as a primary care diagnostic questionnaire in Sweden [19]. They carried out a study in six Swedish primary healthcare centers in two districts, one rural and one semi-urban, to validate the HSCL-25 against the PSE-9 and establish a cut-off.

A cut-off of 1.55 indicated a patient at risk, but a cut-off of 1.75 specified that the patient needed treatment. A cut-off of 1.75 gave a sensitivity of 73%, a specificity of 76%, a Positive Predictive Value (PPV) of 58% and a Negative Predictive Value (NPV) of 86% [19].

The HSCL-25 is not currently used by French GPs, but is a potentially promising tool. The aim of this project, inspired by the Nettlebladt study, was to determine

- the efficiency of the HSCL-25 French version (F-HSCL-25) in French general practice by comparing it with the Present State Examination-9 French version (F-PSE-9), a widely accepted semi-structured clinical interview used extensively in psychiatry [16]
- the internal validity and its dimensions.

## Method

### Study design

A quantitative cross-validation study of the F-HSCL-25 in an adult French general practice population was carried out by the research team of the Soins primaires, Santé Publique, Registre des tumeurs de Bretagne Occidentale (EA 7479 SPURBO). It was a comparative, non-inferiority, multi-centered, survey. The study team constituted of two physician researchers, three GP trainees specifically trained in psychiatric assessment using the PSE-9 and using the CATEGO algorithms [16], a psychiatrist, a statistician, a GP research network of 20 GPs, a Data Manager and a Research Coordinator. The psychiatrist of Brest CHRU trained the GP trainees in psychiatric assessment and confirmed the validity of the clinical diagnoses. A multidisciplinary research network supported the study.

The inclusion period was 20 weeks. The duration of participation for each patient was 1 week. The study was conducted between June 2015 and February 2016.

### Participants

The study was carried out in northern Finistère (Brittany, France) in three study centres (family practice offices affiliated to SPURBO). The population was a mix of patients from urban, semi-rural and rural environments. In the waiting room, before their primary care appointment, patients were given a leaflet explaining the study, an F-HSCL-25 scale and a consent form. Participants were recruited spontaneously to ensure the representativeness of the recruited population, after they had read the explanatory notice and completed the F-HSCL-25 (paper version).

#### Inclusion criteria

The patients needed to be adults (over 18 years). Patients had to give their written informed consent to participate. They completed the F-HSCL-25 self-assessment questionnaire and submitted it to the study team.

#### Exclusion criteria

To avoid possible cases of puerperal depression, which requires specific management, women with a reported pregnancy were not included in the study [20][21][22]. Also excluded were adults consulting for administrative purposes, patients known to be schizophrenic or having related disorders and patients requiring emergency care.

### Sample size

Patients were placed in an HSCL+ group or an HSCL- group according to their scores: F-HSCL-25 score ≥1.75 (or HSCL+) and F-HSCL-25 score <1.75 (or HSCL-). To obtain two balanced groups for final analysis, one in two patients in the HSCL+ group were randomly administered an PSE-9 interview, and one in sixteen patients in the HSCL- group were administered an F-PSE-9. This process ensured the two groups were as comparable as possible.

The delay between interview and inclusion had to be between one week and one month in order to prevent bias in the results of the PSE-9 interview. This was particularly important where an F-HSCL-25 score of ≥1.75 initiated treatment by the GP.

These ratios assume a prevalence of depression between 5% and 12% which gives reasonable precision in estimating diagnostic performance [5]. At least 45 patients were needed per group to ensure a power of 80% in order to detect a difference of at least 50% in the number of people with a PSE-9+ result in the HSCL+ group, compared with 20% with a PSE-9+ result in the HSCL- group.

This required the recruitment of 810 patients. To compensate for those lost to follow-up, the research team decided to include 1100 patients.

The randomization was achieved independently, via computer software, excluding any human intervention in the selection.

### Ethics

The entire study obtained the ethical agreement of the PPC (Protection of Persons Committee). Patients had to give their written, ethical consent to participate. (ID RCB: n°2014-A01790-47; reference CPP: CPP Ouest VI 872;  N° Clinical Trial.gov: NCT02414711).

All patients with a score of ≥ 1.75 were informed by the investigating physician, that they could be depressed, in order to initiate the necessary care with their GPs, according to ethical principles and the ethical consent form.

### Statistical analysis

The data was analysed by the Data Management Unit of the Brest University Hospital (Brest CHRU), and the statistical analyses were carried out using SAS software version 9.4 and R version 3.2.0. The tests were carried out with an alpha risk of 5%.

Descriptive Analysis: Quantitative variables are expressed as means, standard deviations, 25, 50 and 75 quantiles, minimum and maximum values. Qualitative variables are expressed as ratios and percentages.

Comparative Analysis: Univariate comparisons were carried out using relevant standard tests (Student’s, Wilcoxon’s, chi-squared and Fisher’s tests).

External HSCL-25 validation: the PPV and NPV were directly calculated, according to formulas based on a contingency table, but this was not possible for sensitivity and specificity. Due to a different artificial sampling step for the PSE-9 positive/negative patient groups, the prevalence was not respected. The corrected proportions for the contingency table were calculated, taking into account the number of positive/negative patients and the number of included patients. The whole calculation is in S2 Appendix. For each parameter, 95% confidence intervals were computed by bootstrap using R library boot.

To determine the dimensions of the F-HSCL-25 scale, a Principal Component Analysis (PCA) was carried out on the distribution of the items [23][24][25]. To simplify the interpretation of results, a Varimax rotation was performed [26]. The internal consistency (Cronbach’s Alpha calculation) of each dimension was calculated.

## Results

### Clinical and demographic features

**Fig 1:**
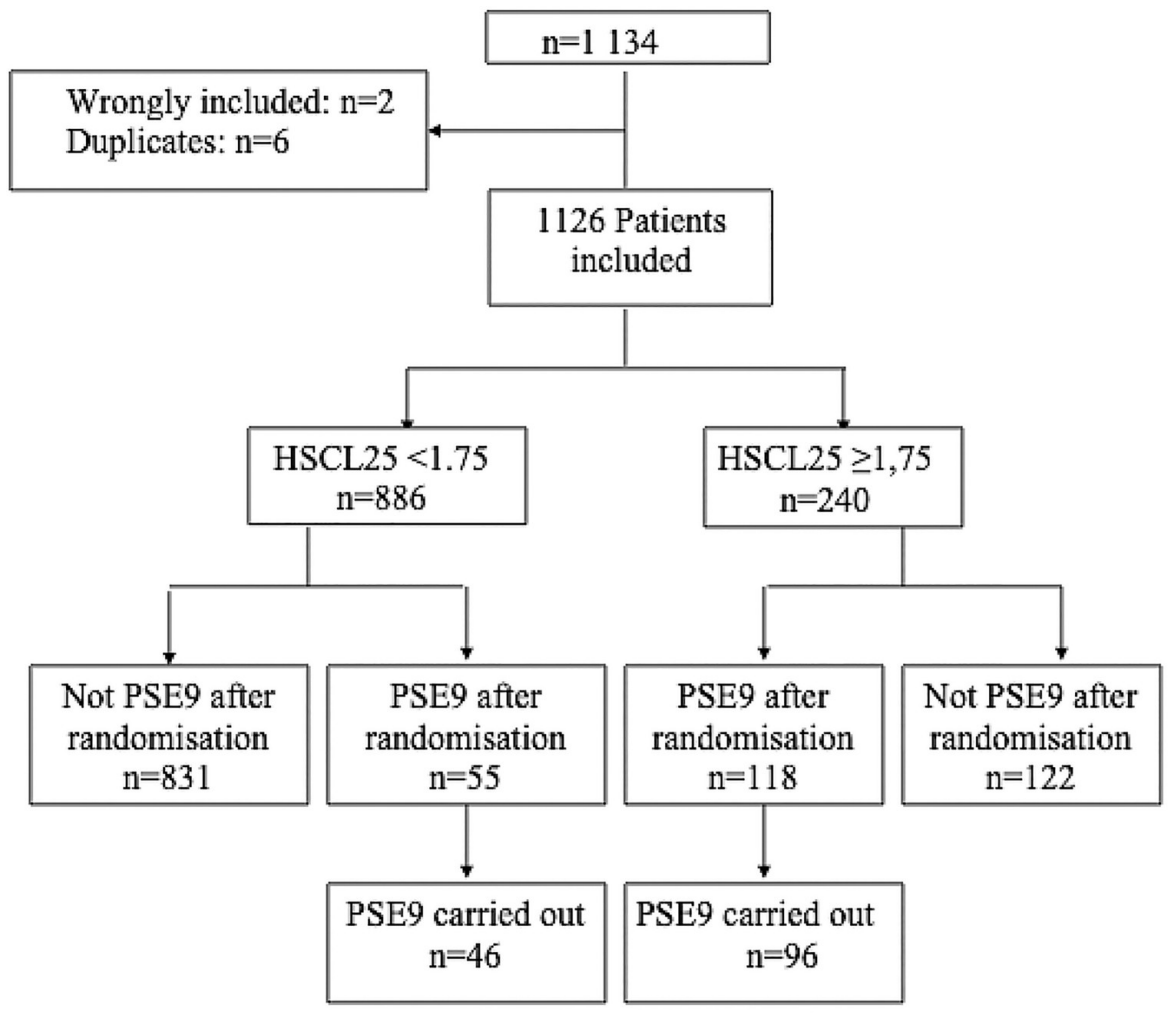
Flow diagram.

The Flow diagram (Fig 1) shows the number of included patients who had filled in the HSCL-25, whether they were randomised to the PSE-9 group or not, and also shows those who took the PSE-9.

1134 patients were selected: 2 patients were wrongly included (a pregnant patient and a patient with related disorders) and 6 were duplicates.

1126 patients filled in the HSCL-25 questionnaire. The two groups were created.

**HSCL- group:**
- 886 patients were randomized according to a ratio of 1/16.
- 831 did not take the PSE-9 test, the study ended for these patients

**HSCL+ group:**
- 240 patients were randomized according to a ratio of 1/2.
- 122 did not take the PSE-9 test, the study ended for these patients.

### Prevalence pitfall

A prevalence established by the F-HSCL-25 of 21.3% was identified among patients consulting their GPs. At the beginning, the sample size was calculated according to prevalence between 5% and 12%. This led to some imbalance in the number of PSE-9 assessments being carried out in the HSCL+ and HSCL- groups.

The study included 1126 French outpatients consulting their GP. Patients were aged between 18 and 94 years. The median age was 59 years and the gender ratio (F/M) was 1.49, Table 1.

**Table 1:** Patients’ characteristics.

Variable	Overall Population	Group F-HSCL-25 <1.75	Group F-HSCL-25 ≥1.75	Inter-group comparisons*
	(N = 1126)	(N = 886)	(N = 240)	
Age				t(408.53) = 3.66
Mean +/- SD	55.62 +/- 18.4	56.61 +/- 18.6	51.98 +/- 17.0	
Median (q1-Q3)	59 (42–70)	61(42–72)	53(38–66)	
min-max	18–94	18–94	19–91	P<0.001
Gender				Chi(1) = 25.24
Male	452 (40.14%)	390 (44.02%)	62 (25.83%)	
Female	674 (59.86%)	496 (55.98%)	178 (74.17%)	P<0.001

*Inter-group comparisons obtained by Student t test for quantitative variables and Chi^2^ test for qualitative variables

### Contingency

55 patients in the HSCL- group had to take the PSE-9. 9 were lost to follow-up; 118 patients in the HSCL+ group had to take the PSE-9. 22 were lost to follow-up. Contingency data are expressed in Tables 2 and 3 (Tables A and B in S2 Appendix).

**Table 2:** Contingency table HSCL-25/PSE-9, before prevalence correction.

		**PSE-9**		**TOTAL**
		**« Positive »**	**« Negative »**	
**HSCL-25**	**« Positive »**	67	29	96
	**« Negative »**	6	40	46
**TOTAL**		73	69	142

**Table 3:** Estimated contingency table HSCL-25/PSE-9, after prevalence correction.

		**PSE-9**		**TOTAL**
		**« Positive »**	**« Negative »**	
**HSCL-25**	**« Positive »**	21.12 (15%)	9.14 (6%)	30.26
	**« Negative »**	14.57 (10%)	97.16 (68%)	111.73
**TOTAL**		35.69	106.3	142

### Outcomes

According to a prevalence of 21.3% (including prevalence corrections) and a cut-off of 1.75, accuracy data gave the following efficiency features expressed in Table 4.

**Table 4:** Efficiency features.

	Value	IC95% *
**PPV**	69.79	[60.61–78.98]
**NPV**	86.96	[77.22–96.69]
**Sensitivity**	59.17	[43.59–80.85]
**Specificity**	91.40	[88.49–94.06]

*Obtained by bootstrap

### Internal consistency and dimensional structure

According to the PCA, two dimensions are retained:

- a group 1 (corresponding to a "depression" dimension), items 11–19 and 21–24,
- a group 2 (corresponding to an "anxiety" dimension), items 1–10, 20 and 25.

Items related to anxiety are items 1–10; items related to depression are items 11–25, in the HSCL-25 original version (Table 5).

**Table 5:** Weight of items in each dimension according to factor analysis.

	1 factor selected	2 factors selected	
ITEM	Axis 1 Depression and Anxiety combined	Group 1 (Axis 1) Depression	Group 2 (Axis 2) Anxiety
Item 1	0.54	0.19	*0*.*60*
Item 2	0.58	0.25	*0*.*58*
Item 3	0.50	0.17	*0*.*56*
Item 4	0.69	0.27	*0*.*73*
Item 5	0.51	0.08	*0*.*67*
Item 6	0.53	0.19	*0*.*57*
Item 7	0.72	0.36	*0*.*67*
Item 8	0.48	0.22	*0*.*47*
Item 9	0.71	0.38	*0*.*63*
Item 10	0.67	0.38	*0*.*57*
Item 11	0.60	**0.57**	0.27
Item 12	0.69	**0.58**	0.39
Item 13	0.56	**0.43**	0.36
Item 14	0.45	**0.46**	0.16
Item 15	0.67	**0.64**	0.30
Item 16	0.73	**0.70**	0.33
Item 17	0.75	**0.69**	0.35
Item 18	0.55	**0.61**	0.15
Item 19	0.68	**0.65**	0.30
Item 20	0.68	0.39	*0*.*58*
Item 21	0.68	**0.75**	0.19
Item 22	0.63	**0.71**	0.16
Item 23	0.68	**0.69**	0.25
Item 24	0.54	**0.48**	0.28
Item 25	0.59	0.36	*0*.*47*

In bold: items related to depression; In italic: items related to anxiety

The Cronbach’s Alpha was calculated for a one-dimensional and a two-dimensional tool (Table 6).

**Table 6:** The F-HSCL-25 Cronbach’s Alpha.

DIMENSION	CRONBACH’S ALPHA
**1 dimension analysis**	
Item axis 1 Depression and Anxiety combined	0,93
**2 dimensions selected according to PCA**	
Group 1 axis 1 Depression (items 11–19 et 21–24)	0,89
Group 2 Axis 2 Anxiety (items 1–10, 20 et 25)	0,87

Since the questionnaire contains 25 elements, it can have up to 25 dimensions.

The first point of the graph indicates the amount of information provided by the first dimension. This point has a high eigenvalue.

The second point provides information on the second dimension. This point has a lower eigenvalue than the previous one.

**Fig 2:**
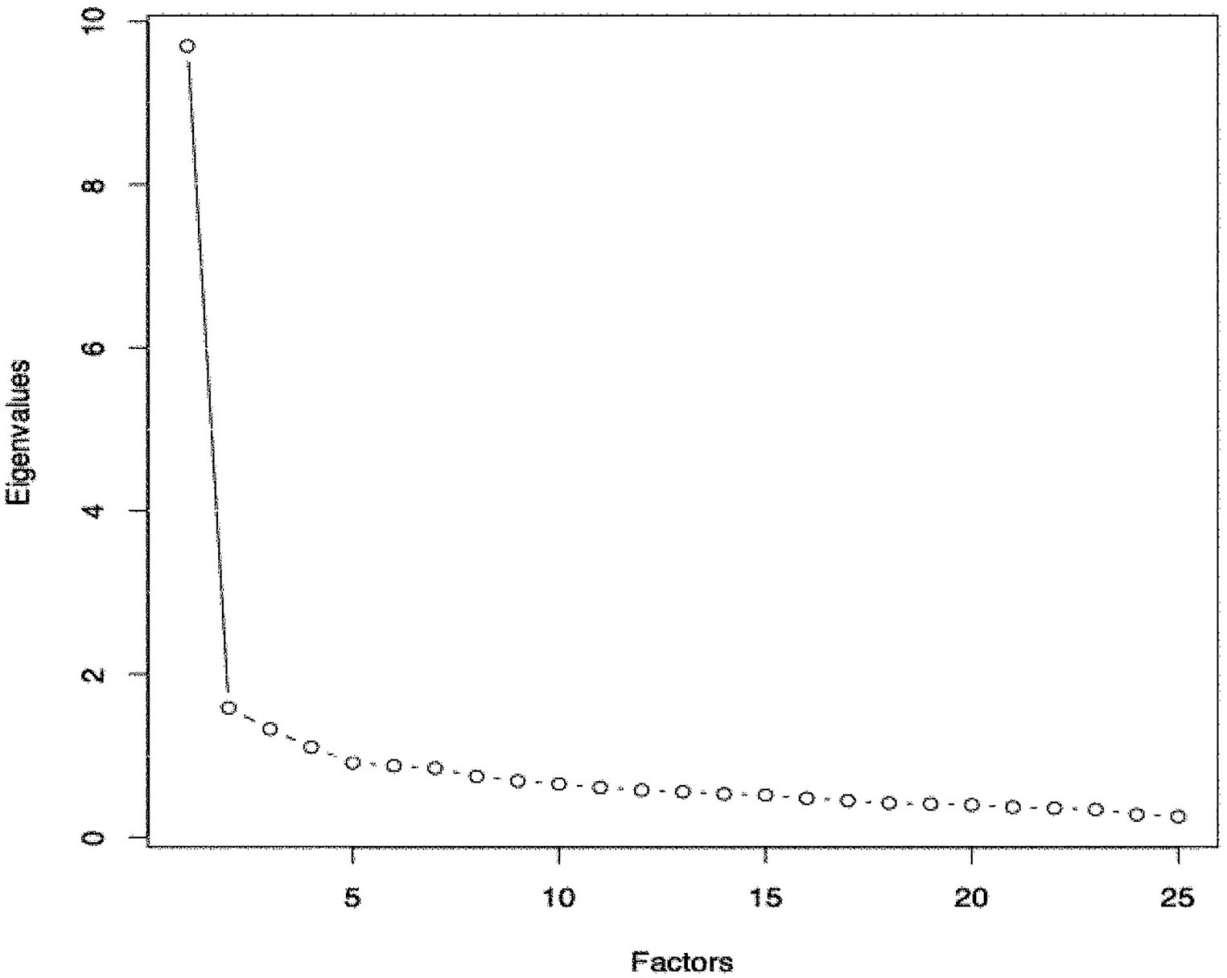
Diagram of eigenvalues.

The important difference between the first and second dimensions and the significant break in the curve shows that F-HSCL-25 is a one dimension tool (Fig 2), anxiety and depression are intimately combined.

## Discussion

### Main findings

F-HSCL-25 adequately assessed major depression. It successfully demonstrated a capacity to facilitate the recognition of a major depressive episode with a PPV greater than 60%. The specificity of 91% indicated efficiency in identifying significant depression in primary care settings. It is a useful first-line ergonomic diagnostic tool with a low number of false positive patients. The GPs’ high depression diagnosis specificity, combined with this tool’s efficiency in excluding non-depressive patients with a low margin of error, may serve to identify patients with depressive symptoms much more rapidly.

F-HSCL-25 is a reliable one-dimensional tool. It was a consistent scale, since the elements (depression and anxiety) converged towards the same result [27][28]. The Cronbach’s Alpha of 0.93 (upper 0.7) proved to have a high internal consistency with outpatients [29][30][31].

### General discussion

Compared to the study by Nettlebladt, [19][20][21] this study resulted in a lower sensitivity (59% versus 76%), it had a higher specificity (91% versus 73%). The prevalence of conspicuous psychiatric morbidity was lower (21% versus 33%). Previous studies showed similar results in terms of sensitivity and specificity [19].

A cut-off point of 1.75 was established for case definition in the original English version. According to Nettlebladt & al., choosing a lower cut-off point (1.55) tended to raise the sensitivity (89%), but also gave higher false positives (43%), making it less accurate. Screening capacity is improved at the expense of diagnostic capacity. Due to the average sensitivity rate and the high specificity in the French study, the HSCL-25, with a cut-off point of 1.75, is valuable in diagnosing patients who require a specific treatment for depression.

The use of a different randomization for each group: a ratio of 1/2 for HSCL+ group, a ratio of 1/16 for HSCL- group, could explain the differences in terms of prevalence, sensitivity and specificity compared with Nettelbladt’s study. Nevertheless, the difference in randomization ratios allowed us to balance the number of F-PSE-9 patients in our groups more closely.

A more recent Swedish study by Lundin & al. also examined the concordance between the HSCL-25 scale score and the DSM-IV depression and anxiety disorders using a well-known semi-structured psychiatric interview (SCAN) as a criterion standard [32]. It differs from the previously mentioned studies due to its large sample (8613 patients recruited) based on a general population although not a medical outpatients’ population. It found that both the depression and anxiety scales of HSCL-25 performed well in detecting their respective DSM-IV disorders. A combined (global) scale also performed efficiently. Nettlebladt’s diagnostic performance, with the cut-off >1.75, showed a higher sensitivity (67.1%), a lower specificity (78.4%), a much weaker PPV (29.8%) but a better NPV (94.6%) than this survey. Our results are comparable with the survey by Lundin and are better than the survey by Nettelbladt.

These results merit comparison with the external validity data of other tools for use in primary care. HSCL-25 like the HADS, is built along two axes: anxiety and depression. HADS has been tested in primary care. It has a higher sensitivity and specificity compared to HSCL-25 (between 0.84 and 0.96) [33]. The ergonomics of this tool seemed more complex to the researchers who preferred the HSCL-25 [12].

The PHQ-9 has a sensitivity between 0.77 and 0.88 and a specificity between 0.88 and 0.94 [34][35]. It is built on the PRIME-MD, not the DSM. The tools are numerous; researchers will make their choices according to their objectives. Systematic reviews or Meta analyses would then be very useful [36].

HSCL-25 originated from HSCL-90. HSCL-90 focused on core and peripheral anxiety and depression symptoms. The HSCL-25 combined anxiety and depression assessment [37][38][39]. Considering the 25 items independently, and as invariant, and considering the sub-parts separately could be a pitfall. As demonstrated by the principal component analysis, items can change from **indicating** depression to **indicating** anxiety and vice versa. F-HSCL-25 should be considered as a global tool to assess the intensity of anxiety and depression [40].

In primary care research, the multiplicity of users requires the use of tools with high consistency [27]. Using self-questionnaires allows independent rating by GPs. On the other hand, this specificity focused on patients makes it impossible to analyse results provided by inter-examiners. The Cohen’s Kappa calculation was not possible with this self-questionnaire. Its format is not an interview-style conducted by an examiner, as is the case for example for HADS. [41].

### Strengths

The strength of this study and its relevance for GPs lies in the fact it is specifically set in primary care.

Several types of data quality procedures were followed which increased the reliability of the results, including the appointment of a designated DRCI data manager at the Brest CHRU. Furthermore, the expertise of the stakeholders in the team was balanced to make data collection secure. A stratified randomization was used to ensure both satisfactory statistical power and affordable logistics.

Women accounted for 60% of the sample. The mean age was 59 years. These sample features were comparable to other studies in primary care settings (51 years). The sample characteristics are close to European population-based norms which make it feasible to generalize from these results [2].

### Weaknesses

A prevalence of 21.3% was identified among patients consulting their GPs. At the beginning of the study, the sample size was calculated according to a prevalence of 5% to 12% in the general population. This study focused on a population which consulted the GP [42]. This prevalence was close to that in Hesbacher’s study, but lower than those in Nettelbladt’s and Golberg’s studies [8,30,34]. Overestimation of the prevalence is possible due to the internal structure of the HSCL-25. This may occur when anxiety and depression are considered separately; however, it is consistent when anxiety and depression are combined [42,43]. In research, the high NPV and specificity, which enable us to eliminate the false positives, also limit this bias. Therefore, physicians should take this into account in their clinical work. To increase the sensitivity, the HSCL-25 could be combined with a screening tool such as the PHQ-2 [43]. With Brittany currently having the highest rate of suicide in France, it is possible that the depression rate in this region may be higher than in France as a whole [44]. This difference has been taken into account in the statistical analysis. The number of subjects was reassessed during the study because of the unexpected distribution of the patients in the two groups. The number of subjects necessary to guarantee the statistical power of the study did not depend on this prevalence but on the minimum number of patients placed in each subgroup. This imbalance does not influence the statistical power of the global study. There were 31 (17.9%) lost to follow-up out of the 173 subjects chosen to take the PSE-9 assessment. Other patients replaced them in accordance with the original randomization method. The protocol had entirely anticipated this bias by allowing for 20% to be lost to follow-up.

The electronic observation book (eCRF) guaranteed the anonymity of the subjects, allocating them a number and keeping only the first two letters of the surname and first name and the date of birth. The eCRF allowed monitoring and enabled traceability of the study. A research assistant checked the validity and consistency of the information between the paper questionnaires and the eCRF. All collected data were compiled into a numeric database. At the end of the study, all information was checked one last time and the database was frozen before statistical work to prevent any information bias.

All responses collected during the PSE-9 interviews were retrospectively analysed under the psychiatrist’s supervision to avoid misinterpretations and to limit any confusion bias.

### Implications

The F-HSCL-25 performs well in detecting symptoms of depression in French primary care and similarly, given its high specificity, provides suitable estimates for clinical research purposes. Its high internal consistency guarantees its reliability. Its design and ease of use guarantee its feasibility.

The characteristics of F-HSCL-25 make this tool suitable for research, rooted as it is in daily practice. Its possible use by healthcare professionals with basic diagnostic skills in mental health could be an advantage in multidisciplinary research. As this study was carried out among unselected adult patients, further investigations could examine the performance of the HSCL-25 in its French version. This could include specific samples in primary care, for example, in student populations or in elderly patients, as has already been carried out in Norway and in Sweden respectively [45].

## Conclusion

Anxiety and depression show considerable overlap in primary care. The structure of the F-HSCL-25, which closely combines anxiety and depression, makes it suitable for general practice and makes it an it efficient tool for detecting a major depressive episode.

This useful, first-line ergonomic and reliable diagnostic tool, combined with GPs’ high diagnosis specificity, could serve to identify patients with symptoms of anxiety and depression much more rapidly.

Its validation throughout Europe, in its translated version, with the same study design, could be of significant epidemiological importance and facilitate the development of more collaborative research within Europe on the subject of depression.

## Supporting information

S1 Appendix HSCL-25 Original version / HSCL-25 French version. (DOCX)

S2 Appendix Calculation of the F-HSCL-25 predictive values. (DOCX)

## Abbreviations

Brest CHRU: Centre Hospitalier Régional Universitaire de Brest
CIC: Centre d’Investigation Clinique
CPP: Comité de Protection des Personnes
DRCI: Délégation à la Recherche Clinique et à l’Innovation
DSM IV / V: Diagnostic and Statistical Manual of Mental Disorders 4^th^ / 5^th^ Edition
DUMG: Département Universitaire de Médecine Générale
eCRF: electronic case report
F-HSCL-25: French version HSCL-25
GPs: General Practitioners
HSCL-25: Hopkins Symptom Checklist—25 items
NPV: Negative Predictive Value
PHQ-2: Patient Health Questionnaire 2 items
PPV: Positive Predictive Value
PSE-9: Present State Examination in its 9^th^ version
Se: Sensitivity
Sp: Specificity
SPURBO = EA 7479 SPURBO: Soins primaires, Santé Publique, Registre des Tumeurs de Bretagne Occidentale
