# Supplementary material for: The French version of the HSCL-25 has now been validated for use in primary care
Source: PLoS One. 2019 Apr 4;14(4):e0214804. doi: 10.1371/journal.pone.0214804 (PMC6448853; doi:10.1371/journal.pone.0214804)
Supplement: S1 Appendix — (DOCX) [file pone.0214804.s001.docx]

S1 Appendix: HSCL-25 Original version / HSCL-25 French version

| **ITEMS** | **HSCL-25 ORIGINAL VERSION** | F-HSCL-25 |
| --- | --- | --- |
| **N°** |  |  |
|  | Choose the best answer for how you felt over the past week | Veuillez choisir la réponse qui décrit le mieux comment globalement vous vous sentiez toute la semaine dernière |
| 1 | Being scared for no reason | Vous avez peur sans raison |
| 2 | Feeling fearful | Vous vous sentez effrayé |
| 3 | Faintness | Vous avez une sensation d’étourdissement |
| 4 | Nervousness | Vous vous sentez nerveux |
| 5 | Heart racing | Vous avez l'impression que votre cœur bat anormalement vite |
| 6 | Trembling | Vous avez la sensation de trembler |
| 7 | Feeling tense | Vous vous sentez tendu |
| 8 | Headache | Vous avez des maux de tête |
| 9 | Feeling panic | Vous vous sentez paniqué |
| 10 | Feeling restless | Vous vous sentez agité |
| 11 | Feeling low in energy | Vous manquez d’énergie |
| 12 | Blaming oneself | Vous ressentez une sensation de culpabilité |
| 13 | Crying easily | Vous pleurez facilement |
| 14 | Losing sexual interest | Vous ressentez un désintérêt pour la vie sexuelle |
| 15 | Feeling lonely | Vous avez une sensation de solitude |
| 16 | Feeling hopeless | Vous vous sentez désespéré |
| 17 | Feeling blue | Vous avez le cafard |
| 18 | Thinking of ending one’s life | Vous avez pensé à mettre fin à votre vie |
| 19 | Feeling trapped | Vous vous sentez pris au piège |
| 20 | Worrying too much | Vous vous inquiétez trop |
| 21 | Feeling no interest | Plus rien ne vous intéresse |
| 22 | Feeling that everything is an effort | Tout est un effort pour vous |
| 23 | Worthless feeling | Vous avez le sentiment d’être bon à rien |
| 24 | Poor appetite | Vous avez perdu l’appétit |
| 25 | Sleep disturbance | Votre sommeil est perturbé |
