# Supplementary material for: The French version of the HSCL-25 has now been validated for use in primary care
Source: PLoS One. 2019 Apr 4;14(4):e0214804. doi: 10.1371/journal.pone.0214804 (PMC6448853; doi:10.1371/journal.pone.0214804)
Supplement: S2 Appendix — (DOCX) [file pone.0214804.s002.docx]

S2 Appendix: Calculation of the F-HSCL-25 predictive values

**Table A. Contingency table HSCL-25/PSE-9, before prevalence correction**

|  | | **PSE-9** | | **TOTAL** |
| --- | --- | --- | --- | --- |
|  | | **« Positive »** | **« Negative »** |  |
| **HSCL-25** | **« Positive »** | 67 (69.79%) | 29 (30.21%) | 96 |
|  | **« Negative »** | 6 (13.04%) | 40 (86.96%) | 46 |
| **TOTAL** |  | 73 | 69 | 142 |

We could calculate PPV and NPV directly from the contingency table, according to the following formulas:

PPV = TP / (TP + FP) =67 / (67 + 29) =0.70

NPV = TN / (TN + FN) =40 / (40 + 6) =0.87

However, the sampling step was artificial. It was determined by the protocol to improve the feasibility of the study, as 1/16 (HSCL-) and 1/2 (HSCL +) patient. The prevalence is not respected.

We could not apply the contingency table directly, according to the formulas for Se and Sp

**Corrective formulas to obtain Se and Sp**

The probability of the test being positive or negative from the contingency table should be calculated as follows:

The number of positive tests (HSCL ≥ 1.75) divided by the number of patients included: P (HSCL +) =(HSCL +) / N

The number of negative tests (HSCL <1.75) divided by the number of patients included: P (HSCL-) =(HSCL-) / N

N =1126

P(HSCL+) =(HSCL+) / N =240 / 1126 =0.21

P(HSCL-) =(HSCL-) / N =886 / 1126 =0.79

Now we are able to calculate the corrected proportions for the contingency table:

Proportion of True Positive =PPV * P (HSCL +) =0.70*0.21 =0.15

Proportion of True Negative =NPV * P (HSCL-) =0.87*0.79 =0.68

Proportion of False positive =(1-PPV) * P (HSCL +) =(1-0.7)*0.21 =0.06

Proportion of False Negative =(1-NPV) * P (HSCL-) (1-0.87)*0.79 =0.10

**Table B. Estimated contingency table HSCL-25/PSE-9, after prevalence correction**

|  | | **PSE-9** | | **TOTAL** |
| --- | --- | --- | --- | --- |
|  |  | **« Positive »** | **« Negative »** |  |
| **HSCL-25** | **« Positive »** | 21.12 (15%) | 9.14 (6%) | 30.26 |
|  | **« Negative »** | 14.57 (10%) | 97.16 (68%) | 111.73 |
| **TOTAL** |  | 35.69 | 106.3 | 142 |

The corrected number on the contingency table can then be calculated by multiplying by the number of patients who have passed the PSE (142 outpatients).

Then directly apply the calculation formulas:

Se = TP / (TP + FN) =21.12 / (21.12+35.69) =0.59

Sp = TN / (TN + FP) =97.16 / (97.16 + 9.14) =0.91

The calculation of the NPV and the PPV from the initial or modified contingency table were, of course, identical.

This could be expressed concisely and applied rapidly by using the following corrective formulas directly:

*Se = PPV * P(HSCL+) / [P(HSCL+) * PPV] + [P(HSCL−) * (1−NPV)]*

*Sp = NPV * P(HCSL−) / [P(HSCL+) * PPV] + [P(HSCL−) * (1−NPV)*

Se = Sensitivity; Sp = Specificity; P: Prevalence; PPV = Positive Predictive Value; NPV = Negative Predictive Value; P(HSCL+) = Patient HSCL+ frequency; P(HSCL$-$) = Patient HSCL$-$ frequency
